# Estimation of right lobe graft weight for living donor liver transplantation using deep learning-based fully automatic computed tomographic volumetry

**DOI:** 10.1038/s41598-023-45140-0

**Published:** 2023-10-18

**Authors:** Xiaopeng Yang, Seonyeong Park, Seungyoo Lee, Kyujin Han, Mi Rin Lee, Ji Soo Song, Hee Chul Yu, Jae Do Yang

**Affiliations:** 1 School of Global Entrepreneurship and Information Communication Technology, Handong Global University, Pohang, 37554 Republic of Korea; 2 Department of Surgery, Jeonbuk National University Medical School and Hospital, Jeonju, 54907 Republic of Korea; 3 Research Institute of Clinical Medicine, Jeonbuk National University, Jeonju, 54907 Republic of Korea; 4 Biomedical Research Institute, Jeonbuk National University Hospital, Jeonju, 54907 Republic of Korea; 5 Department of Radiology, Jeonbuk National University Medical School and Hospital, Jeonju, 54907 Republic of Korea

**Keywords:** Gastrointestinal system, Liver diseases

## Abstract

This study aimed at developing a fully automatic technique for right lobe graft weight estimation using deep learning algorithms. The proposed method consists of segmentation of the full liver region from computed tomography (CT) images, classification of the entire liver region into the right and left lobes, and estimation of the right lobe graft weight from the CT-measured right lobe graft volume using a volume-to-weight conversion formula. The first two steps were performed with a transformer-based deep learning model. To train and evaluate the model, a total of 248 CT datasets (188 for training, 40 for validation, and 20 for testing and clinical evaluation) were used. The Dice similarity coefficient (DSC), mean surface distance (MSD), and the 95th percentile Hausdorff distance (HD95) were used for evaluating the segmentation accuracy of the full liver region and the right liver lobe. The correlation coefficient (CC), percentage error (PE), and percentage absolute error (PAE) were used for the clinical evaluation of the estimated right lobe graft weight. The proposed method achieved high accuracy in segmentation for DSC, MSD, and HD95 (95.9% ± 1.0%, 1.2 ± 0.4 mm, and 5.2 ± 1.9 mm for the entire liver region; 92.4% ± 2.7%, 2.0 ± 0.7 mm, and 8.8 ± 2.9 mm for the right lobe) and in clinical evaluation for CC, PE, and PAE (0.859, − 1.8% ± 9.6%, and 8.6% ± 4.7%). For the right lobe graft weight estimation, the present study underestimated the graft weight by − 1.8% on average. A mean difference of − 21.3 g (95% confidence interval: − 55.7 to 13.1, *p* = 0.211) between the estimated graft weight and the actual graft weight was achieved in this study. The proposed method is effective for clinical application.

## Introduction

Living donor liver transplantation (LDLT) has become an effective treatment option for patients with end-stage liver disease^1,2^. Accurate graft weight estimation is vital to the safety of both recipients and donors in LDLT. An appropriately sized graft is essential for the success of LDLT. For the recipient, an inadequate graft could cause small-for-size syndrome^3–10^, whereas an excessively large graft could result in large-for-size syndrome^11^. For the donor, an insufficient remnant liver volume after graft harvest could lead to postoperative liver dysfunction^12,13^.

The right lobe graft weight has been estimated directly from the graft volume measured via computed tomography (CT) volumetry^1^ or indirectly using formulas that convert graft volume to graft weight^1,14–18^. Direct estimation of graft weight from graft volume was based on the assumption that the liver has the same density as water^19,20^; however, the density of the liver is slightly higher than that of water^21^. Thus, graft weight directly estimated from graft volume tended to be larger than actual graft weight measured intraoperatively^15,17,22–26^. The indirect formulas converting graft volume to graft weight can be classified into two groups; one with blood volume included in graft volume^15,16^ and the other with blood volume excluded from graft volume^1,14,17,18^. Formulas from the former group tended to have larger errors than those from the latter group because the graft weight measured intraoperatively excludes the weight of blood^1^.

Graft volume is commonly measured using commercial software in clinical practice. First, the full liver is extracted from CT images either semi-automatically or fully automatically by conventional machine learning algorithms^27–29^ or deep learning algorithms^14,30^. Then blood vessels, including the hepatic vein and portal vein, are extracted and then excluded from the extracted full liver^1^. Next, the liver is manually divided into the left and right lobes based on anatomical landmarks^1,14^. Lastly, the graft volume is calculated from the divided right lobe. The total processing time is ranged from 1.3 to 8 min^1,14^. For example, Park et al. used a commercial software solution which applies a deep learning algorithm for fully automated segmentation of the liver. After they corrected any segmentation errors, they defined the resection plane for the right lobe graft based on the Cantlie line by manually drawing two dividing lines^14^. The entire process of their method is still not fully automatic due to the manual division of the liver into the left and right lobes. Therefore, this study aimed to propose a deep learning-based fully automatic technique for division of the liver into the left and right lobes for right lobe graft weight estimation and evaluate its performance.

## Methods

### Study population

This study was approved by the Institutional Review Board of Jeonbuk National University Hospital (IRB No. 2022-08-001). This study was performed in accordance with the Declaration of Helsinki and participants’ informed consent was waived by the review board of Jeonbuk National University Hospital due to the retrospective nature of this study. We included 248 patients (171 males and 77 females; mean age: 50.0 ± 12.3 years; age range: 18–79 years), with healthy livers at Jeonbuk National University hospital from January 2009 to December 2021. Among them, 228 patients were healthy persons for medical checkup, whereas 20 patients were healthy LDLT donors. The 228 patients for medical checkup were randomly split into 188 patients (including 125 males and 63 females; mean age: 52.0 ± 11.0 years; age range: 30–79 years) and 40 patients (including 30 males and 10 females; mean age: 50.0 ± 9.5 years; age range: 32–74 years) for training and validation of the proposed deep learning technique, respectively. The 20 LDLT donors (16 males and 4 females; mean age: 29.5 ± 9.8 years; age range: 18–55 years) were used for test and clinical evaluation of the proposed deep learning technique. All the patients underwent abdominal CT examinations. The LDLT donors had their graft weights measured intraoperatively.

### CT imaging

CT scans were obtained by a 128-row multidetector CT scanner SOMATOM Definition AS+ (Siemens, Forchheim, Germany) using a standard four-phase (non-contrast liver, late arterial phase, portal venous phase, and delayed phase) contrast enhanced imaging protocol. The resulting CT scans have a slice thickness of 1 mm or 3 mm. CT scans of the delayed phase were used in the present study.

### CT volumetric measurement

**Figure 1:**
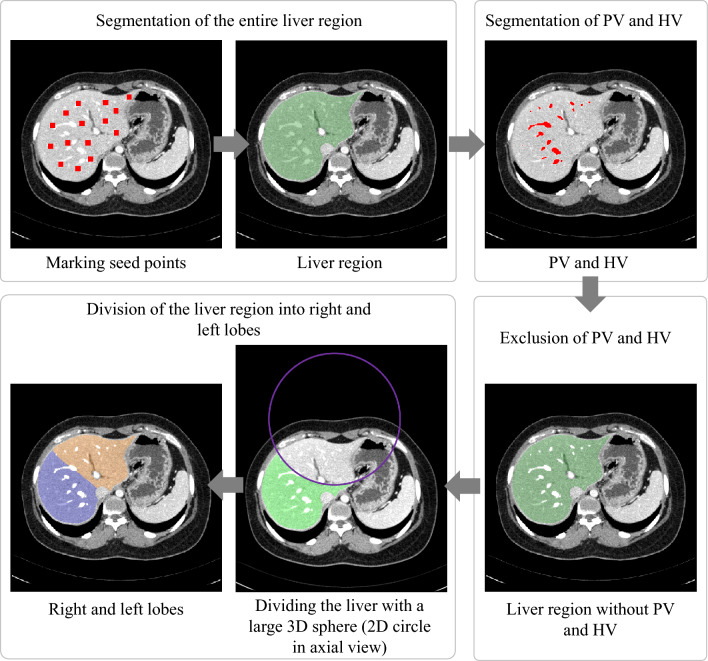
Preparation of the ground truth images of the entire liver region and the left and right liver lobes. PV = portal vein, HV = hepatic vein.

The ground truth images of the entire liver region and the left and right liver lobes were generated by an expert with more than ten years of experience in abdominal imaging using Dr. Liver (Humanopia, Inc., Pohang, Korea), as shown in Fig. 1. First, the entire liver region was semi-automatically segmented from a CT scan by putting seed points over the liver region, with the liver region interactively edited if necessary, in Dr. Liver. Second, blood vessels, including the portal vein, and hepatic vein, were automatically segmented, and excluded from the segmented liver region in Dr. Liver. The segmented liver regions without the portal vein and hepatic vein were used as the ground truth images for whole liver segmentation. Third, the segmented liver region without blood vessels was interactively divided into the left and right lobes in Dr. Liver by following Cantlie’s line going through the center of the inferior vena cava, the middle hepatic vein, and the middle of the gallbladder bed^31^. The classified left and right lobes were used as the ground truth images for the classification of the entire liver region into the left and right lobes. The volume of the segmented liver and that of the classified right lobe were measured using the summation-of-area method^32^.

### Intraoperative graft weight measurement

The liver graft of each LDLT donor was flushed at the back table with histidine-tryptophan-ketoglutarate solution (Custodiol; Köhler Chemie, Als-bach-Hähnlein, Germany) and then trimmed and weighed intraoperatively. The right liver lobes were harvested as liver grafts in the present study.

### Deep learning model

**Figure 2:**
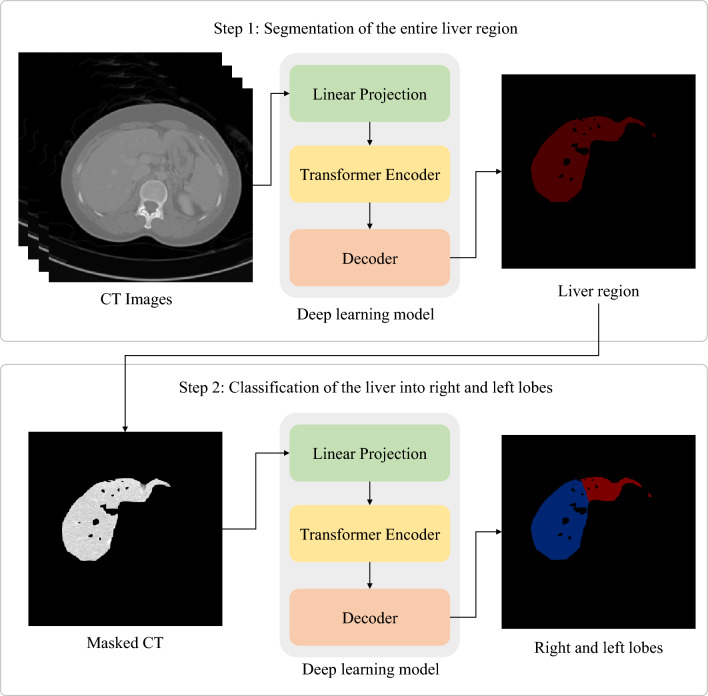
The proposed deep learning-based technique for classifying the liver into the right and left lobes.

As shown in Fig. 2, the proposed method consists of two steps: (1) segmentation of the entire liver region from a CT dataset and (2) classification of the liver into the left and right lobes from a CT dataset masked by the segmented liver region. In both steps, a deep learning model known as UNETR^33^ was used. The UNETR model consists of a transformer encoder to learn contextual information from the embedded input patches and is connected to a convolutional neural network (CNN)-based decoder through a skip connection to predict the segmentation outputs. The datasets were split into training, validation, and test sets with proportions of 76%, 16%, and 8%, respectively. The CT images were resampled into the isotropic voxel spacing of 1.0 mm and then randomly cropped with volume sizes of 32 × 32 × 32. Data augmentation was applied to the training data with a random flip in axial, sagittal, and coronal views. The UNETR models in the two steps were trained using a workstation with two NVIDIA RTX A5000 graphics cards. Both models were trained using the AdamW optimizer^34^ with an initial learning rate of 0.0001 and a decay rate of 0.00001. The model in the entire liver region segmentation step and that in the classification step were trained for 20,000 iterations and 10,000 iterations, respectively. For both models, the transformer-based encoder includes 12 layers and an embedding size of 768. A patch resolution of 16 × 16 × 16 was used. For post-processing, a 3D connected component method and morphological operations including erosion and dilation were used to improve segmentation results. For all datasets, the erosion operation with a radius of one was performed three times followed by three times of dilation with a radius of one. The parameters of the erosion and dilation operations were determined empirically.

### Evaluation: metrics and statistical analysis

The segmentation results of the entire liver region and the right lobe were evaluated using the dice similarity coefficient (DSC), the 95th percentile of the Hausdorff Distance (HD95), and the mean surface distance (MSD). The DSC is used to measure the voxel overlap between the prediction (P) and the ground truth (G). The HD and MSD are used to measure the surface-based distance between the surfaces of P (S_P_) and G (S_G_), whereas HD measures the maximum surface distance. The three metrics are defined as follows:

1 $$\mathrm{DSC}=\frac{2\left|P\cap G\right|}{\left|P\right|+\left|G\right|}\times 100\%$$

2 $$\mathrm{HD}\left({S}_{P},{S}_{G}\right)=\mathrm{max}\left(\underset{p\in {S}_{P}}{\mathrm{max}}\underset{g\in {S}_{G}}{\mathrm{min}}\Vert p-g\Vert ,\underset{g\in {S}_{G}}{\mathrm{max}}\underset{p\in {S}_{P}}{\mathrm{min}}\Vert p-g\Vert \right)$$

3 $$\mathrm{MSD}=\frac{1}{\left|{S}_{P}\right|+\left|{S}_{G}\right|}\left\{\sum_{p\in {S}_{P}}\underset{g\in {S}_{G}}{\mathrm{min}}\Vert p-g\Vert +\sum_{g\in {S}_{G}}\underset{p\in {S}_{P}}{\mathrm{min}}\Vert p-g\Vert \right\}$$

For the clinical evaluation, a total of 20 donors with CT examinations and graft weights measured intraoperatively (actual graft weights) were included. The graft volume from CT volumetry measured using the proposed deep learning method was converted to the graft weight (predicted graft weight) using the formula graft weight = 206.3 + 0.653 × graft volume^14^. The predicted graft weight was then compared with the actual graft weight measured intraoperatively in terms of the percentage error (PE, %) and percentage absolute error (PAE, %). PE is defined as the ratio of the difference between the predicted graft weight and the actual graft weight to the actual graft weight. PAE is the absolute value of PE. Pearson’s correlation test was performed to obtain the coefficient of the correlation between the predicted graft weight and the actual graft weight. The paired *t*-test was used to test whether or not the predicted graft weight and the actual graft weight differed significantly from each other. All statistical tests were performed using Minitab version 18 (Minitab, LLC, State College, PV) at *p* < 0.05.

## Results

### Entire liver region segmentation

**Figure 3:**
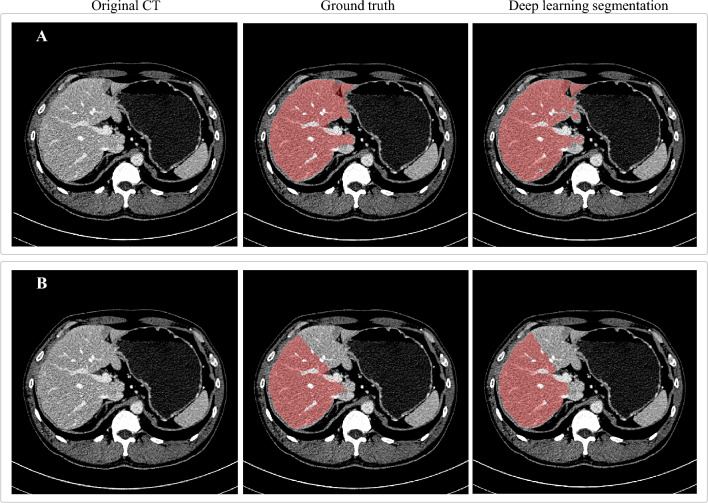
Illustration of segmentation results using the proposed deep learning-based technique. (**A**) The entire liver region. (**B**) The right liver lobe.

The mean ± standard deviation (SD) values of DSC, MSD, and HD95 for the entire liver region segmentation were 95.9% ± 1.0%, 1.2 ± 0.4 mm, and 5.2 ± 1.9 mm, respectively (Table 1). Figure 3A illustrates the entire liver region segmentation result of a patient from the test dataset.

**Table 1:** Evaluation of segmentation results for the entire liver region and the right lobe by the proposed deep learning-based method with a test set (*n* = 20).

Liver part	DSC (%)	MSD (mm)	HD95 (mm)
Entire liver	95.9 ± 1.0 (93.5–97.4)	1.2 ± 0.4 (0.7–2.3)	5.2 ± 1.9 (3.0–11.7)
Right lobe	92.4 ± 2.7 (83.0–95.0)	2.0 ± 0.7 (1.2–4.6)	8.8 ± 2.9 (5.7–16.2)

*DSC* dice similarity coefficient, *MSD* mean surface distance, *HD95* the 95th percentile Hausdorff distance.

### Right lobe segmentation

The mean (± SD) values of DSC, MSD, and HD95 for the segmentation of the right lobe were 92.4% ± 2.7%, 2.0 ± 0.7 mm, and 8.8 ± 2.9 mm, respectively (Table 1). Figure 3B illustrates the right lobe segmentation result of a patient from the test dataset.

### Clinical evaluation

**Figure 4:**
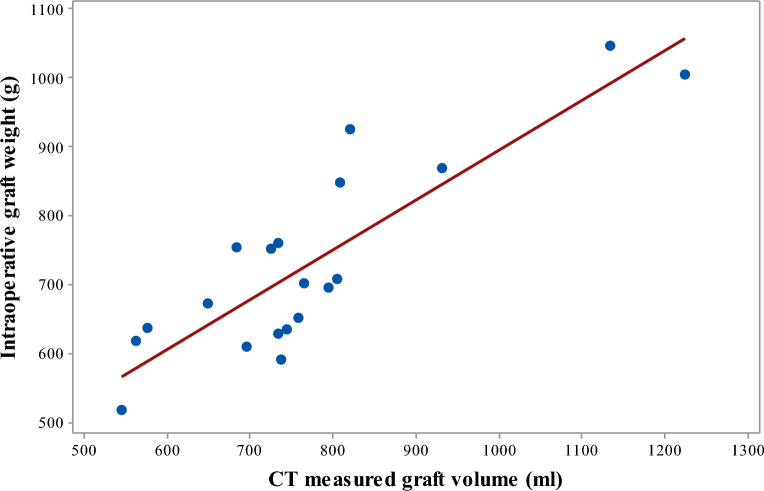
The plot of intraoperatively measured graft weight to CT measured graft volume using the proposed deep learning-based method.

**Figure 5:**
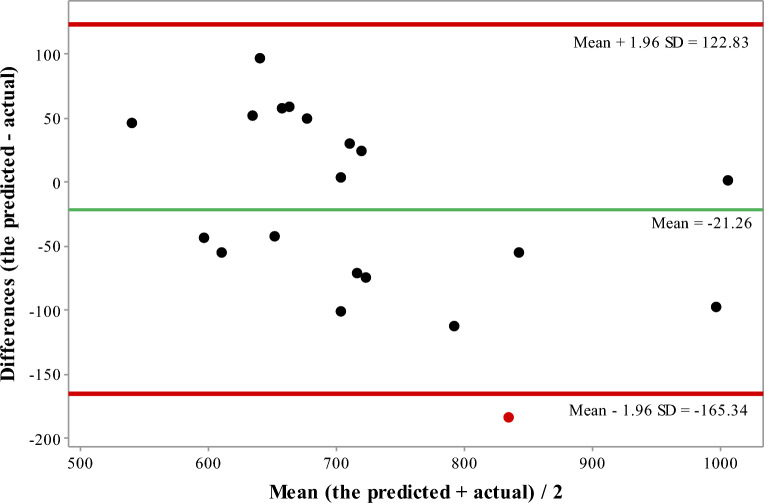
Bland–Altman plot of the difference between the predicted graft weight using the proposed deep learning-based method and the intraoperatively measured graft weight.

The clinical evaluation results are shown in Table 2. The mean (± SD) graft volume was 770.2 ± 163.8 ml. The mean (± SD) intraoperative graft weight was 730.5 ± 138.0 g. Figure 4 shows the plot of the intraoperatively measured graft weight to CT measured graft volume. The mean (± SD) predicted graft weight was 709.2 ± 107.0 g. The mean (± SD) PE in graft weight estimation was − 1.8% ± 9.6%. The mean (± SD) PAE in graft weight estimation was 8.6% ± 4.7%. The coefficient of the correlation between the predicted graft weight and the actual graft weight was 0.859 (*p* < 0.001). The paired *t*-test showed that the predicted graft weight did not significantly differ from the actual graft weight (*t*(19) = ‒1.29, *p* = 0.211). Figure 5 shows the Bland–Altman plot of the difference between the predicted graft weight using the proposed deep learning-based method and the intraoperatively measured graft weight.

**Table 2:** Clinical evaluation results for graft weight estimation by the proposed deep learning-based method with a test set (*n* = 20).

CT-measured graft volume (ml)	Intraoperatively measured graft weight (g)	Predicted graft weight (g)	PE (%)	PAE (%)
770.2 ± 163.8 (544.2–1223.5)	730.5 ± 138.0 (516.0–1045.0)	709.2 ± 107.0 (561.7–1005.2)	− 1.8 ± 9.6 (− 19.9–16.2)	8.6 ± 4.7 (0.1–19.9)

*PE* percentage of error, *PAE* percentage of absolute error.

## Discussion

In the present study, we proposed a fully automatic technique for estimating the right lobe graft weight using deep learning-based CT volumetry. The proposed method consists of three steps: (1) segmentation of the entire liver region, (2) classification of the liver into the left and right lobes, and (3) estimation of the right lobe graft weight from right lobe graft volume with a volume-to-weight conversion formula. The proposed method achieved higher accuracy in right lobe graft weight estimation (correlation coefficient to the actual graft weight = 0.859; PAE = 8.6% ± 4.7%), compared to that reported in Kwon et al.’s study^35^ (correlation coefficient to the actual graft weight = 0.807; PAE = 9.0% ± 8.7%). Kwon et al.’s method requires intensive manual intervention for CT volumetry using a commercial medical software application, whereas the proposed method is effective and fully automatic. The high accuracy of the proposed method is due to the high accuracy of the proposed the deep learning method in liver segmentation and left and right lobe classification and the volume-to-weight conversion formula. Furthermore, the exclusion of blood vessels from the segmented liver region contributes to the high accuracy of the proposed method as well because the graft is intraoperatively weighed after the blood in the liver is drained^1^. For segmentation of the liver, average DSC was 95.9% using the UNETR model in this study. In recent studies^36–38^, average DSC was ranged from 93.08 to 95.72% for segmentation of the liver on the Synapse dataset^39^ using various deep learning-based methods. The UNETR model we used incorporates a transformer encoder and a CNN-based decoder and has been proved to be promising for medical image segmentation^33^. Liver segmentation is still challenging due to the false inclusion of some organs such as the heart and spleen for some cases where the boundaries between the liver and other organs are severely blurry. For right lobe graft weight estimation, the present study underestimated the graft weight by − 1.8% on average. A mean difference of − 21.3 g (95% confidence interval: − 55.7 to 13.1, *p* = 0.211) between the estimated graft weight and the actual graft weight was achieved in this study. Recently, Buijk et al. conducted a systematic review and meta-analysis which included 31 studies for comparing estimated graft volume and actual graft weight^40^. For the right liver graft, they reported that the 31 existing studies overestimated the graft volume by 2.99% on average for manual volumetry. A mean difference of 34.0 g (95% confidence interval: 11.85–56.11, *p* = 0.003) was obtained for manual volumetry. Through comparison, our deep learning-based fully automatic method outperformed the existing studies on average.

For the first time, we proposed to use a deep learning-based method throughout the entire process of right lobe graft weight estimation. Especially, we proposed a deep learning-based method for fully automatic division of the liver into the left and right lobes. Park et al.^14^ claimed that they applied deep learning in right lobe graft weight estimation. However, they only applied deep learning to the segmentation of the entire liver region but not to the classification of the liver into the left and right lobes. Then, they manually classified the liver into the left and right lobes. In our study, the classification of the liver into the left and right lobes was fully automatically performed using deep learning as well. Therefore, our method does not require any manual intervention by the users.

Our study has several limitations. First, it was a single-center study. Though Yang et al.^1^ established a graft weight formula using data from a single medical center and proved that the formula was still accurate by a cross validation using data from a different medical center, it is better to collect more data from different centers to assess the performance of our method. Second, we used CT scans from the delayed phase in the present study. We need to extend our method to the cases from other phases like the portal venous phase. Third, our study was limited by the exclusion of patients with liver masses and cirrhosis. Fourth, the volume-to-weight conversion formula used in the present study tended to underestimate the graft weight. The formula we used was not built with our data and it is linear, which could cause the underestimation. However, we haven’t found any higher-order or non-linear models. We need to establish our own formula by considering higher-order or non-linear models for a more accurate estimation of graft weight after collecting more data. We will address these issues in our future studies.

In conclusion, we proposed a deep learning-based fully automatic method for the entire right lobe’s graft weight estimation process. We evaluated our method in terms of accuracy and clinical practice. The proposed fully automatic method has been proven accurate in clinical practice.

## Data Availability

The datasets generated or analyzed during the study are available from the corresponding author on reasonable request.
